# Preparation of Thermochromic Vanadium Dioxide Films Assisted by Machine Learning

**DOI:** 10.3390/nano14131153

**Published:** 2024-07-06

**Authors:** Gaoyang Xiong, Haining Ji, Yongxing Chen, Bin Liu, Yi Wang, Peng Long, Jinfang Zeng, Jundong Tao, Cong Deng

**Affiliations:** School of Physics and Optoelectronics, Xiangtan University, Xiangtan 411105, China

**Keywords:** machine learning, magnetron sputtering, energy-saving material, VO_2_(M), extreme gradient boosting

## Abstract

In recent years, smart windows have attracted widespread attention due to their ability to respond to external stimuli such as light, heat, and electricity, thereby intelligently adjusting the ultraviolet, visible, and near-infrared light in solar radiation. VO_2_(M) undergoes a reversible phase transition from an insulating phase (monoclinic, M) to a metallic phase (rutile, R) at a critical temperature of 68 °C, resulting in a significant difference in near-infrared transmittance, which is particularly suitable for use in energy-saving smart windows. However, due to the multiple valence states of vanadium ions and the multiphase characteristics of VO_2_, there are still challenges in preparing pure-phase VO_2_(M). Machine learning (ML) can learn and generate models capable of predicting unknown data from vast datasets, thereby avoiding the wastage of experimental resources and reducing time costs associated with material preparation optimization. Hence, in this paper, four ML algorithms, namely multi-layer perceptron (MLP), random forest (RF), support vector machine (SVM), and extreme gradient boosting (XGB), were employed to explore the parameters for the successful preparation of VO_2_(M) films via magnetron sputtering. A comprehensive performance evaluation was conducted on these four models. The results indicated that XGB was the top-performing model, achieving a prediction accuracy of up to 88.52%. A feature importance analysis using the SHAP method revealed that substrate temperature had an essential impact on the preparation of VO_2_(M). Furthermore, characteristic parameters such as sputtering power, substrate temperature, and substrate type were optimized to obtain pure-phase VO_2_(M) films. Finally, it was experimentally verified that VO_2_(M) films can be successfully prepared using optimized parameters. These findings suggest that ML-assisted material preparation is highly feasible, substantially reducing resource wastage resulting from experimental trial and error, thereby promoting research on material preparation optimization.

## 1. Introduction

Vanadium dioxide (VO_2_) is a typical transition metal oxide with over ten crystalline phases (A, B, D, P, M, etc.) [1,2,3,4,5,6]. However, only VO_2_(M) undergoes a metal-insulator transition at 68 °C, drawing significant attention from researchers [7,8,9]. When the temperature rises to the critical temperature, VO_2_(M) rapidly transforms from a low-temperature monoclinic structure to a high-temperature tetragonal structure, resulting in a sudden change in the optical and electrical properties of VO_2_. VO_2_(M) has a wide range of applications in various fields, including optoelectronic switches [10,11], smart windows [12,13], military camouflage [14,15,16,17], and spacecraft thermal control [18,19]. Thus, the preparation of high-purity VO_2_(M) films has long been a key concern in VO_2_ research, as it determines the effectiveness of applications of VO_2_ film.

Magnetron sputtering, a widely employed physical vapor deposition method, is considered a promising technique for manufacturing VO_2_(M) films due to its high uniformity and strong adhesion to the substrate [20,21]. However, the preparation of high-purity VO_2_(M) through magnetron sputtering requires precise control of sputtering parameters, such as sputtering power, substrate temperature, and the ratio of the oxygen to argon flow rate [22]. Therefore, the precise control of these deposition conditions is extremely important for achieving high-purity VO_2_(M) films. However, the complex management of deposition conditions during VO_2_ preparation introduces uncertainty in the sedimentary results. Thus, it is vital to explore and optimize the preparation process of the VO_2_(M) films. In the field of materials science, significant efforts from predecessors have led to the accumulation of abundant experimental and computational data for material preparation, containing numerous experimental parameters and conditions. Consequently, it is urgently needed to manage this vast dataset for robust data handling methods. Machine learning (ML), a typical data-driven pattern research method [23,24], fulfills the requirements for a big data statistical analysis. It is a powerful tool for learning from training existing datasets, enabling the extraction of underlying patterns and facilitating regression or classification on previously unseen data. ML has been widely applied in various fields of materials science, such as discovering new compounds or molecules with desired properties [25], material synthesis [26,27,28], material structure design [29,30], and material structure and performance prediction [31,32,33,34]. Owing to its potent capabilities, its application in aiding the preparation of high-purity VO_2_(M) materials holds significant prospects.

In this paper, multi-layer perceptron (MLP), support vector machine (SVM), random forest (RF), and extreme gradient boosting (XGB) were implemented to train the preparation dataset of VO_2_ obtained from laboratory experiments and the literature. Each model was evaluated based on indicators such as specificity, accuracy, and recall to optimize the final deposition process. Additionally, a feature importance analysis was conducted on the dataset to explore the influence of different deposition conditions on the results. The results revealed that XGB demonstrated the highest classification performance on this dataset, achieving a prediction accuracy of 88.52% on the test dataset. The XGB model can effectively instruct the preparation of high-purity VO_2_(M). This paper offers insights into the use of ML methods for aiding the targeted generation of VO_2_ materials, while the proposed prediction model introduces novel approaches for optimizing the research of material preparation technologies.

## 2. Results and Discussion

### 2.1. Optimized Synthesis Frame Design

**Figure 1:**
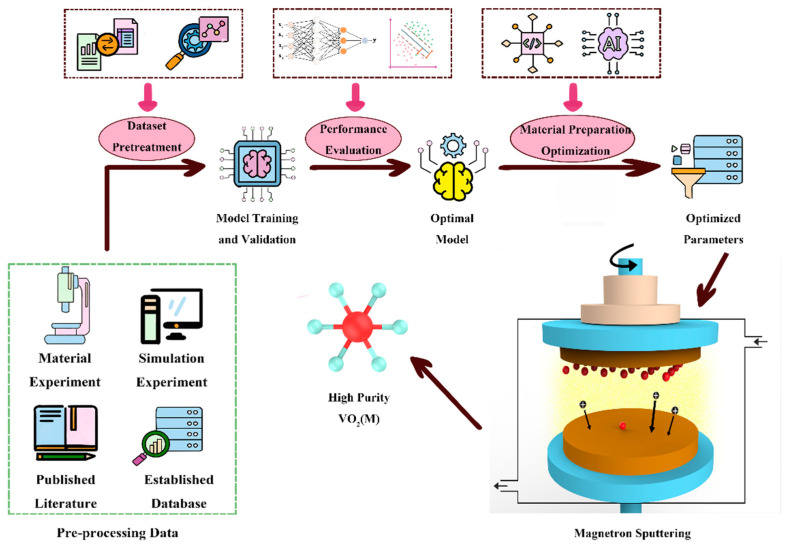
Machine learning optimization framework for VO_2_(M) magnetron sputtering preparation.

During the experimental procedures, it is usually desired to achieve an effective preparation of the target material under predetermined deposition conditions. However, under varied experimental conditions, fixed parameters may fail to yield optimal results. Thus, to avoid unnecessary experimental errors and minimize costs, it is crucial to determine a model capable of adjusting experimental conditions and predicting results accurately. In this article, an ML-assisted approach for depositing VO_2_ films by magnetron sputtering was presented (Figure 1). The conventional magnetron sputtering method entails introducing specific proportions of O_2_ and argon gas into a high-vacuum environment, where the target material undergoes oxidation and is subsequently deposited onto the substrate surface via magnetron sputtering to produce VO_2_(M). In this study, ML can aid in optimizing the deposition parameters for VO_2_(M) films. Before sputtering, predictive models were employed to forecast deposition parameters, thereby determining the likelihood of achieving pure-phase VO_2_(M) under these conditions and using the predicted probability as a benchmark for adjusting comprehensive parameters. The high probability of sedimentation parameters in the experiments significantly diminished the uncertainty of the results and minimized resource waste caused by trial and error. 

### 2.2. Dataset Establishment

Data preparation is a fundamental aspect of optimizing material preparation. In this article, a dataset was constructed, including 203 experimental data points from laboratory experiments and the relevant literature. The crystal structure of VO_2_ was identified by comparing the X-ray diffraction (XRD) data with the JCPDS standard card, serving as the foundation for data acquisition [35]. Table 1 presents the experimental parameters utilized in the preparation of VO_2_(M) via magnetron sputtering, mainly including sputtering power, substrate temperature, ratio of the oxygen to argon flow rate, reaction pressure, target material, and substrate. 

**Figure 2:**
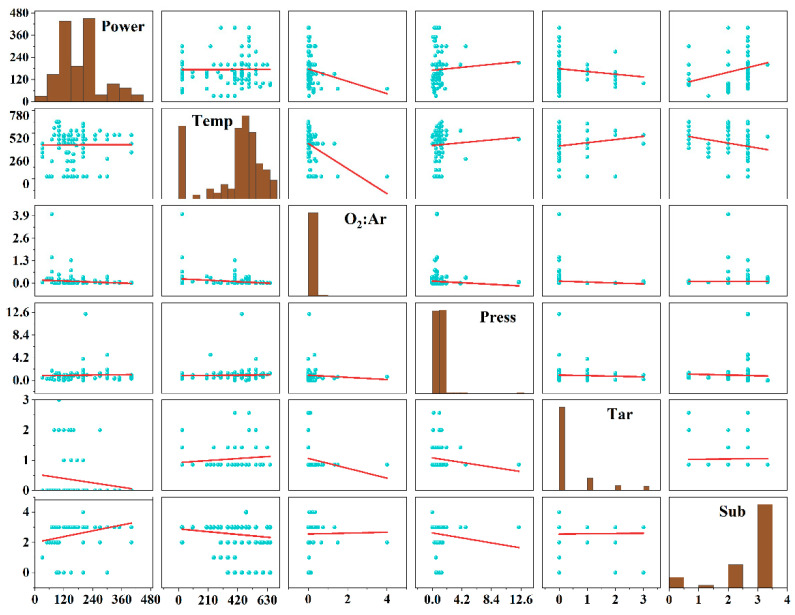
Scatter plot matrices of collected data. The 0, 1, 2, and 3 of Tar represent V, V_2_O_5_, VO_2_, and V_2_O_3_, respectively, and the 0, 1, 2, 3, and 4 of Sub represent Al_2_O_3_, NaCa glass, Si, SiO_2_, and stainless steel, respectively.

**Figure 3:**
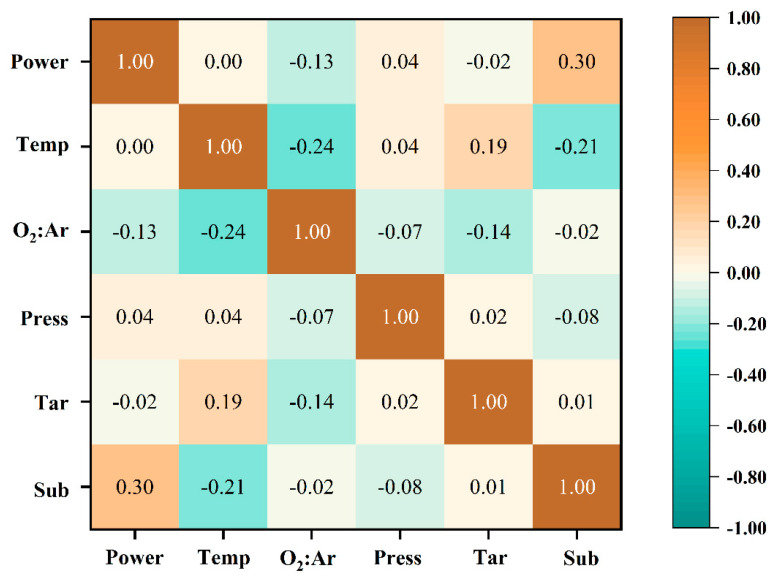
Correlation analysis of dataset infrared heat map.

Before model training, the dataset was preprocessed, including encoding the string data and assigning numerical values of “1” and “0” to pure-phase VO_2_(M) and non-VO_2_(M), respectively. The scatter plot matrix is a visual aid used to analyze the relationships between multiple variables, as illustrated in Figure 2. A Pearson correlation coefficient analysis was employed to assess the degree of interdependence among variables in the dataset. The correlation between the six features obtained is shown in Figure 3. The positive and negative values in the figure represent positive and negative correlations between features, respectively. Based on the analysis results, the correlation coefficients between the selected features approached 0, indicating a high degree of independence among each feature, thereby reducing the data redundancy that arises from highly correlated data and the problem of feature weight allocation during model training.

### 2.3. Classification Model

Following dataset establishment and preprocessing, it was necessary to train four ML models (MLP, XGB, SVM, and RF) to accurately predict experimental results. Before inputting the dataset, it needed to be segmented, with 75% randomly allocated as the training set and the remaining 25% as the validation set. After building the model framework, it was essential to adjust the hyperparameters of the model [36], search for hyperparameters of each model through a grid search, and then fine-tune based on validation set performance, aiming to determine the optimal hyperparameters and ensure the best predictive performance. Additionally, to prevent overfitting in model predictions, ten-fold cross-validation was conducted for each model. Subsequently, the test set was utilized to comprehensively assess the performance of each model. Eventually, the optimal model was chosen based on the evaluation results and employed to guide the optimization of the magnetron sputtering process. This model can predict material preparation results and minimize the uncertainty of deposition experiments.

### 2.4. Model Selection and Performance Evaluation

**Figure 4:**
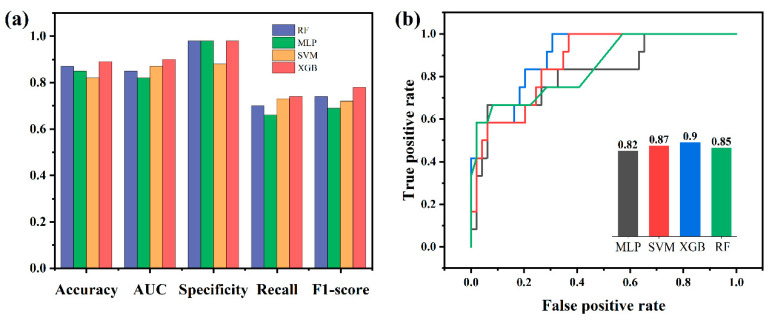
(**a**) Bar chart of model performance comparison; (**b**) ROC curve of each model.

To optimize magnetron sputtering, a thorough performance evaluation was conducted on the ML model, and the optimal model based on a dataset with high confidence was selected. This study utilized four ML algorithms—MLP, XGB, SVM, and RF—for training based on the original dataset. Previous studies have demonstrated the widespread application and effectiveness of these four models in the optimization of material preparation, particularly on small datasets [27,37,38]. To identify the conditions for the optimal model, this article assessed six indicators, the receiver operating characteristic curve (ROC), area under the ROC curve (AUC), accuracy, specificity, recall, and F1 score, to evaluate the performance of the four models. Among these indicators, ROC assessed the model’s predictive ability, with the AUC value representing its efficacy. Accuracy measured the percentage of correctly predicted samples, specificity quantified the percentage of correctly predicted negative samples, recall determined the percentage of correctly predicted positive samples, and the F1 score represented the harmonic average of accuracy and recall. The results of the model evaluation are depicted in Figure 4a. Overall, these results indicated rapid convergence of all ML models without encountering overfitting or underfitting issues, and they exhibited outstanding predictive capabilities.

**Figure 5:**
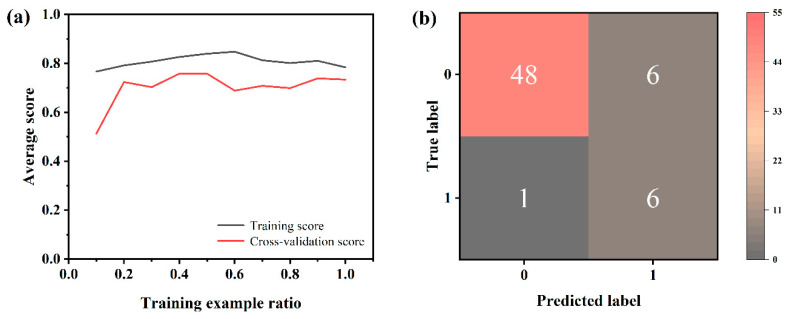
(**a**) Learning curve of XGB model; (**b**) confusion matrix of XGB model.

Among the four trained models, the XGB model exhibited the highest prediction accuracy, reaching 88.52%, which is higher than previous relevant reports [39,40], followed by the RF model at 86.89%. The F1 score was a comprehensive evaluation indicator of accuracy and recall. Comparing the F1 scores of the four models, XGB still achieved the highest score of 0.78. Regarding other evaluation indicators, XGB still maintained the highest recall rate as well as specificity, which were 0.74 and 0.98, respectively. The ROC curve was considered one of the most important evaluation tools for classification models. Usually, the closer the curve is to the upper left corner, the better its classification performance. Figure 4b illustrates the ROC curves of the four models, demonstrating that the XGB model exhibited the curve closest to the upper left corner, with the highest AUC value of 0.90 among them. Based on the above, this study further investigated the optimization of preparation pathways using XGB models. Figure 5a displays the learning curve of the XGB model. As the sample size increased, the model scored steadily on both the training and validation sets, demonstrating the feasibility of using the XGB model without overfitting problems. The confusion matrix is obtained according to the dataset and prediction results, as shown in Figure 5b, and the specificity and sensitivity can be calculated from the matrix, showing that the XGB model has a good performance.

This discovery indicated a significant correlation between the prediction results of the XGB model and the experimental results reported in the published literature. In order to evaluate the predictive performance of the XGB model, the experimental conditions on the preparation of VO_2_ by magnetron sputtering were validated in the recent literature. The prediction results under various experimental conditions are shown in Table 2. Among these conditions, the first three conditions have been confirmed to successfully prepare pure-phase VO_2_(M), while the latter two conditions cannot. These findings indicated a significant correlation between the prediction results of the XGB model and the experimental results reported in the published literature.

### 2.5. Material Synthesis Pathway Optimization

**Figure 6:**
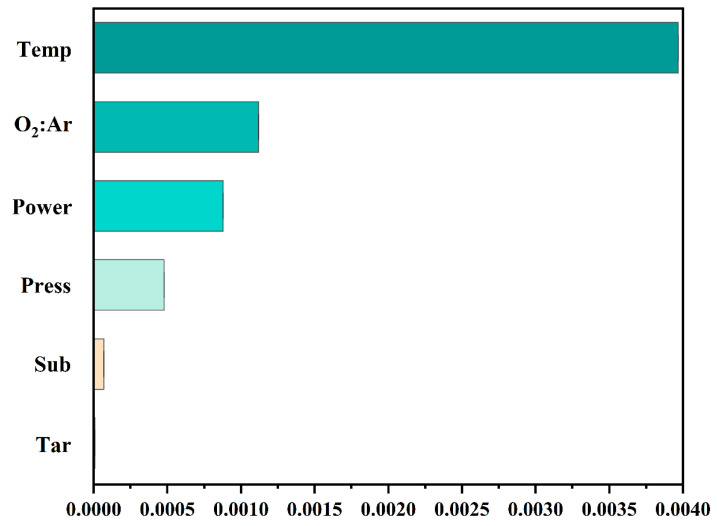
The SHAP value for each feature.

To optimize the feature parameters of the preparation process, the Shapley Additive exPlanning (SHAP) method was used to extract the importance of features from the six feature parameters. SHAP is a tool for interpreting model outputs. By analyzing the impact of each feature parameter on the output, the sum of Shapley values (contributions) of all feature parameters can be obtained, which is the magnitude of importance [41]. Figure 6 demonstrates that substrate temperature (Temp) had the most significant impact on the purity of VO_2_(M), followed by the ratio of the oxygen to argon flow rate (O_2_:Ar) and sputtering power (Power), whereas substrate type (Sub) and target type (Tar) had the least impact. To optimize the range of feature parameters, a dataset containing 6,375,000 virtual experimental condition data points was generated. The XGB model is employed to predict the probability of successfully preparing VO_2_(M) from these data. Subsequently, the data with a probability greater than 90% were filtered out, resulting in 1,527,400 high-success-rate data points. The range of values for each feature parameter is shown in Table 3. By selecting the characteristic parameter values within this range, the preparation purity of VO_2_(M) can be optimized to a certain extent, accelerating the controllable preparation speed of the materials.

### 2.6. Model Verification

**Figure 7:**
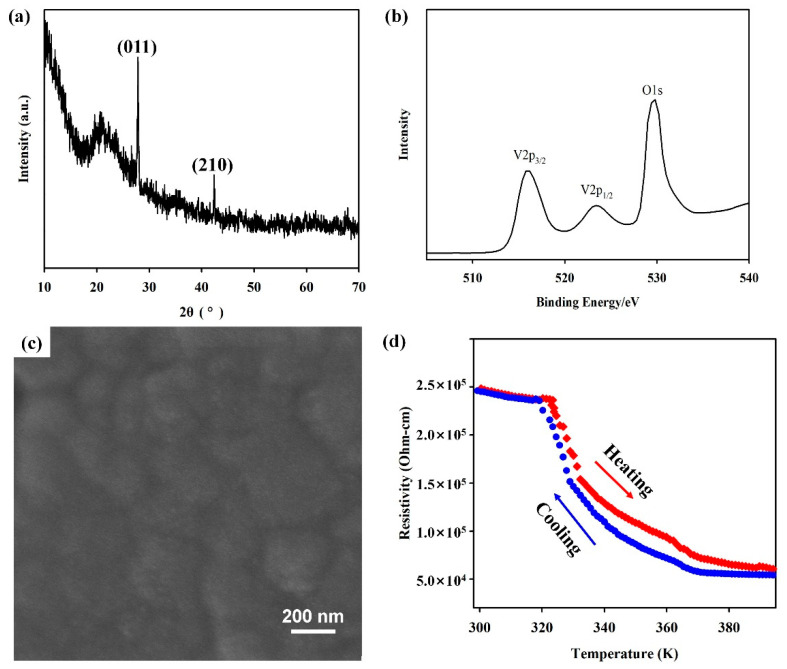
(**a**) XRD pattern; (**b**) XPS spectrum; (**c**) SEM image; (**d**) resistivity vs. temperature of VO_2_(M) prepared with magnetron sputtering.

Furthermore, under the guidance of the XGB model, a set of experimental parameters with high success rates were selected to verify the reliability of the model. The specific experimental conditions are a sputtering power of 150 W, substrate temperature of 550 °C, ratio of the oxygen to argon flow rate of 1.6, reaction pressure of 0.4 Pa, target material of vanadium metal, substrate of SiO_2_, and deposition time of 2 h. According to the prediction of the XGB model, the success rate under these experimental conditions was 93.61%. Under these conditions, magnetron sputtering experiments were conducted, and the products were analyzed using the Bruker D8 advanced X-ray diffractometer. The obtained XRD patterns are shown in Figure 7a. The main diffraction peaks at 2θ degrees of 27.8° and 42.3° corresponded to the (011) and (210) orientations of the VO_2_(M) phase, respectively. The high-resolution XPS spectrum of VO_2_(M) is shown in Figure 7b. The spectrum of V_2p_ can be resolved into two independent peaks, where the V_2p3/2_ peak was 515.8 eV, corresponding to the valence state of V^4+^ ions in VO_2_. The O_1s_ peak at 530.0 eV is usually attributed to the O^2−^ in the sample, which is consistent with the previous results [42,43]. Combined with the XRD results, it can be further determined that the VO_2_(M) was obtained. Figure 7c represents the SEM image of the obtained VO_2_(M) films via magnetron sputtering. The film surface was composed of spherical-like grains from ~80 nm to ~260 nm, with typical grain size of ~180 nm. Due to the transition characteristics of VO_2_ from the low-temperature semiconductor phase to high-temperature metal phase, its resistance changes by nearly two orders of magnitude during heating and cooling between 20 °C and 120 °C (Figure 7d). These experimental findings demonstrate that high-purity VO_2_(M) materials can be successfully prepared with the assistance of the XGB model, validating the ability of the trained ML model to promote the preparation of film materials.

**Table 2:** Prediction of recently published literature parameters using XGB model.

Number	Power (W)	Temp (°C)	O_2_:Ar	Press (Pa)	Tar	Sub	Model Predict	Reference
1	90	650	0.013	1.33	V	Al_2_O_3_	86.23%	Zhang C et al. [44]
2	100	650	0.013	1.33	V	Si	85.50%	Zhang C et al. [45]
3	80	580	0.06	1.00	V	Si	71.47%	Ma X et al. [46]
4	100	500	0.025	1.00	V_2_O_3_	Al_2_O_3_	28.64%	Yang Z et al. [47]
5	200	600	0.05	1.00	V	Si	30.41%	Xiong Y et al. [48]

**Table 3:** Optimizing the range of features. The 0, 1, 2, and 3 of Tar represent V, V_2_O_5_, VO_2_, and V_2_O_3_, respectively, and the 0, 1, 2, 3, and 4 of Sub represent Al_2_O_3_, NaCa glass, Si, SiO_2_, and stainless steel, respectively.

Parameters	Min	Max
Power (W)	110	210
Temp (℃)	500	700
O_2_:Ar	0.1	2.2
Press (Pa)	0.15	2.9
Tar	0 or 1, 2, 3	
Sub	0 or 1, 2, 3, 4	

## 3. Conclusions

Four ML models were selected to train the dataset, and their performance was comprehensively evaluated in this study. Among them, the XGB model exhibited the highest prediction accuracy, reaching 88.52%. The comprehensive parameter space was explored by utilizing the trained XGB model. Then, the range of each feature value was determined with a high probability of success in the preparation process, thus offering experimental guidelines. A feature importance analysis was conducted using the SHAP method, revealing that substrate temperature had a significant impact on the purity of VO_2_(M). Subsequently, experimental verification was performed. The experimental findings confirmed the successful preparation of pure-phase VO_2_(M) films. In conclusion, this study illustrated that machine learning can effectively facilitate the experimental preparation process, thereby minimizing the uncertainty of experimental results and reducing resource consumption associated with trial and error. This study provides methodological guidance for optimizing material preparation, which has enormous potential in advancing materials science.

## Figures and Tables

**Table 1:** Reaction conditions and labeling comparison table.

Mark	Reaction Conditions
Power	The sputtering power of magnetron sputtering
Temp	The temperature of the substrate
O_2_:Ar	The ratio of oxygen to argon flow rate
Press	Magnetron sputtering reaction pressure
Tar	Sputtering target material
Sub	Sputtering reaction substrate

## Data Availability

Data are contained within the article.
